# The Use of Adipose-Derived Stem Cells for the Treatment of Complex Postoperative Enterocutaneous Fistulas: A Preliminary Case Series Study

**DOI:** 10.3390/medicina61122102

**Published:** 2025-11-26

**Authors:** Pietro Fransvea, Valeria Fico, Gilda Pepe, Marta Di Grezia, Gaia Altieri, Giuseppe Tropeano, Sergio Alfieri

**Affiliations:** 1 Department of Medical and Surgical Sciences, Fondazione Policlinico Universitario A. Gemelli IRCCS, 00168 Rome, Italy; pietro.fransvea@policlinicogemelli.it (P.F.); gilda.pepe@policlinicogemelli.it (G.P.); marta.digrezia@policlinicogemelli.it (M.D.G.); gaiaaltieri89@gmail.com (G.A.); tropeano.giuseppe@yahoo.it (G.T.); sergio.alfieri@policlinicogemelli.it (S.A.); 2 Dipartimento di Medicina e Chirurgia Traslazionale, Università Cattolica del Sacro Cuore, 00168 Roma, Italy

**Keywords:** adipose-derived mesenchymal stem cells, enterocutaneous fistulas, regenerative medicine

## Abstract

*Background and Objectives*: Postoperative enterocutaneous fistulas, defined as abnormal communications between the intestinal lumen and the skin, represent one of the most challenging complications following abdominal surgery. Regenerative medicine, particularly through the use of adipose-derived mesenchymal stem cells (ADSCs), has recently emerged as a promising therapeutic option for chronic inflammatory and non-healing conditions. However, most studies have focused on complex perianal fistulas in Crohn’s disease. This prospective, single-center observational study aimed to evaluate the feasibility, safety, and preliminary efficacy of autologous ADSC injection in patients with complex postoperative enterocutaneous fistulas. *Materials and Methods*: Six patients (four males and two females) with persistent postoperative enterocutaneous fistulas were enrolled. Autologous adipose tissue was harvested via lipoaspiration from the abdominal wall or flank and processed in a GMP-certified laboratory to obtain a suspension containing 5–10 million viable ADSCs in 3–5 mL of isotonic solution. ADSCs were injected directly into the fistulous tract under ultrasound guidance, following CT image review. Clinical and radiologic follow-up was performed to assess closure and output reduction. *Results*: Four of the six patients (66.7%) achieved complete fistula closure, with no residual output and radiologic confirmation of healing within 4–12 weeks. One patient (16.7%) demonstrated a significant reduction in fistula output (>80%), while another (16.7%) showed minimal improvement and subsequently required surgical repair at 6 weeks. No complications related to ADSC administration were observed. *Conclusions*: Autologous ADSC therapy appears to be a feasible, safe, and minimally invasive option for managing complex postoperative enterocutaneous fistulas. These encouraging preliminary results—showing complete closure in two-thirds of treated patients—support further investigation through larger, controlled trials to validate these findings and optimize treatment protocols.

## 1. Introduction

Postoperative enterocutaneous fistulas are among the most formidable complications following abdominal surgery, contributing significantly to morbidity, prolonged hospitalization, and increased mortality rates. The annual incidence of enterocutaneous fistula (ECF) is approximately 1.4 to 2.3 per 100,000 people, though some studies report higher rates in specific surgical populations. For instance, a U.S. study found a 1.4% overall incidence in adults with a recent gastrointestinal surgery, with a higher rate for those who had a small bowel resection. These fistulas—often resulting from anastomotic leaks, iatrogenic injury, or infectious complications—create a pathological communication between the intestinal lumen and the skin, resulting in persistent sepsis, fluid loss, malnutrition, and impaired wound healing. Despite advances in surgical technique and perioperative care, complex enterocutaneous fistulas continue to pose a therapeutic challenge.

Conventional management involves a multidisciplinary approach, typically combining sepsis control, nutritional support (parenteral or enteral), wound care, and delayed definitive surgical repair [1,2]. However, the conservative approach alone yields variable success, and reoperation is often necessary. Surgical intervention carries its own risks, especially in patients with hostile abdomens, comorbidities, or previous radiation therapy. The need for reoperation (i.e., a surgical procedure) is higher for fistulas that do not close spontaneously, but the risk of recurrence of the fistula after a definitive surgery is also a significant factor, which can reach as high as 31%.

In recent years, regenerative medicine has emerged as a novel frontier in treating chronic inflammatory and non-healing conditions. Recent advances in tissue engineering and regenerative medicine have highlighted the therapeutic potential of mesenchymal stem cells (MSCs) for gastrointestinal and postoperative complications. In particular, adipose-derived stem cells (ADSCs) exhibit unique biological properties such as high proliferative capacity, potent paracrine signaling, and secretion of growth factors (VEGF, FGF, and TGF-β) that facilitate angiogenesis, epithelialization, and modulation of the local immune response. Several experimental models have demonstrated that ADSCs can enhance wound healing by promoting fibroblast proliferation, extracellular matrix remodeling, and revascularization, thereby supporting tissue closure in chronic wounds and fistulas. These findings provide a strong biological rationale for investigating ADSC therapy in postoperative enterocutaneous fistulas, which are characterized by persistent inflammation, bacterial contamination, and impaired tissue regeneration. Mesenchymal stem cells (MSCs), especially those derived from adipose tissue (ADSCs), have garnered significant attention due to their accessibility, immunomodulatory properties, and potential to enhance tissue repair [3,4,5]. Unlike bone-marrow-derived MSCs, ADSCs can be obtained in higher yield with less donor morbidity [6]. Preclinical and early-phase clinical studies have demonstrated the efficacy of ADSCs in enhancing epithelial regeneration, modulating inflammation, and promoting angiogenesis [7]. In the gastrointestinal field, most clinical applications have focused on complex perianal fistulas in Crohn’s disease [8,9,10]. The ADMIRE-CD trial by Panés et al. demonstrated that local injection of allogeneic expanded ADSCs resulted in a >50% clinical remission at 24 weeks in patients with refractory perianal Crohn’s disease. These results led to regulatory approval for this indication in Europe [11].

Despite these advances, there remains a notable lack of evidence regarding the application of ADSC therapy in postoperative ECFs. This gap is clinically significant, as postoperative fistulas differ from Crohn-related fistulas in their etiology, tissue environment, and inflammatory profile—factors that may influence treatment response. Demonstrating the feasibility and safety of ADSCs in this distinct population could expand their use beyond inflammatory bowel disease, offering a minimally invasive, biologically driven alternative for patients with limited surgical options.

Accordingly, this preliminary case series aims to evaluate the feasibility, safety, and early efficacy of autologous ADSC injection in patients with complex postoperative enterocutaneous fistulas unresponsive to standard care. The present study therefore expands the existing literature by applying autologous ADSCs to postoperative ECFs unrelated to inflammatory bowel disease, providing detailed procedural data, biological characterization of the cell product, and systematic follow-up to assess safety and early efficacy outcomes.

## 2. Materials and Methods

### 2.1. Study Design and Setting

This is a prospective, observational single-center study conducted at high-volume tertiary academic hospital specializing in gastrointestinal and trauma surgery. The study period is between February 2022 and December 2023. The study was conducted in accordance with the Declaration of Helsinki, and the protocol was approved by Ethics Committee of Fondazione Policlinico Universitario A. Gemelli IRCCS (ID 4639) on 7 February 2022. Informed consent for participation was obtained for all subjects involved in the study.

### 2.2. Patient Selection

Patients aged 18–75 years with persistent postoperative enterocutaneous fistulas (ECFs) of more than six weeks’ duration were eligible for inclusion. All patients had failed conservative management, including bowel rest, total parenteral nutrition (TPN), and pharmacologic therapies aimed at reducing intestinal flow. Before inclusion, all patients underwent nutritional and infection status evaluation. Nutritional assessment included serum albumin, prealbumin, and body mass index, ensuring albumin ≥ 2.5 g/dL prior to injection. Infection control was confirmed by negative blood cultures, normalization of C-reactive protein (CRP), and absence of undrained abscess on imaging. The cutoff of serum albumin ≥ 2.5 g/dL was selected as a pragmatic indicator of minimal nutritional adequacy, below which impaired wound healing and increased infection risk are expected. Baseline and week-12 albumin levels were recorded to monitor nutritional recovery over time.

Other inclusion criteria were as follows:

- Fistula output greater than 200 mL/day, as measured by a 24 h collection (typical range among enrolled patients: 200–350 mL/day).
- Presence of a single or complex fistulous tract confirmed by CT-fistulography.
- Nutritional optimization before the procedure, with serum albumin ≥ 2.5 g/dL (used as a threshold for adequate nutritional status).

Exclusion criteria included:

- Inflammatory bowel disease (IBD)
- Malignancy-related fistulas
- Ongoing immunosuppressive therapy
- Severe uncontrolled infection or sepsis
- Pregnancy or breastfeeding

Fistulas were classified as simple (single, short tract, direct communication with the bowel) or complex (multiple tracts, associated abscess cavity, foreign body, or exposure to radiation or mesh), in line with established classifications.

### 2.3. Adipose Harvest and Cell Processing

All procedures were performed under deep sedation with a laryngeal mask airway. All procedures were conducted in a dedicated operating room with laminar airflow. The preparation of adipose-derived mesenchymal stem cells (ADSCs) followed the same standardized protocol previously described by Potenza et al. for complex perianal fistulas in Crohn’s disease [12]. Lipoaspiration was performed using a closed sterile system (Lipokit^®^, Medikan, Seoul, Republic of Korea) to prevent air or bacterial contamination. Approximately 100–150 mL of autologous fat was harvested from the subcutaneous layer of the abdominal wall or flank, using a specially designed 2.1 mm micro-cannula. The adipose tissue was filtered and centrifuged (800× *g* for 10 min) in a GMP-certified laboratory, within 2 h to obtain ADSC. Flow cytometry verified the presence of mesenchymal stem cells surface markers (CD73+, CD90+, CD105+, CD34+, CD45−), with marker positivity > 85% in all samples. A final suspension of 5–10 million viable stem cells in 3–5 mL of isotonic solution was prepared in sterile syringes. Microbiological testing was performed on the final ADSC suspension to confirm sterility before clinical use. The total number of viable cells administered per patient ranged from 5.2 × 10^6^ to 9.7 × 10^6^, depending on yield.

### 2.4. Cell Injection Protocol

Under sterile operating room conditions, ADSCs were injected directly into the fistulous tract. The suspension was administered in multiple small aliquots circumferentially along the tract using a 25-gauge needle to ensure homogeneous distribution of the cells. In all cases, a single injection session was performed; however, additional injections were planned only in case of incomplete response after 12 weeks. The same injection protocol was used for both simple and complex fistulas, without differences in the amount or technique. No concurrent immunosuppressive or anti-inflammatory medications were administered to patients. Antibiotic prophylaxis with Cefazolin and Metronidazole was administered uniformly to all patients; no subject received additional therapeutic antibiotics for ongoing contamination or sepsis at the time of injection.

### 2.5. Follow up and Outcome Measures

Patients were monitored in hospital for 24–48 h to detect adverse reactions after the procedure. They were followed up with outpatient visits at 1, 4 and 12 weeks. The safety follow-up window extended to 12 weeks post-procedure. Prespecified adverse events of special interest included infection, abscess formation, ectopic tissue growth, and thromboembolic events. These were actively solicited at 1, 4, and 12 weeks through clinical assessment and laboratory monitoring.

Parameters assessed included:

- Daily fistula output (mL/24 h)
- Inflammatory Laboratory Markers (C-reactive protein, WBC count)
- Nutritional Laboratory Markers (serum albumin)
- Radiological exams (CT-fistulography or Contrast-enhanced MRI).

Outcomes were defined as follows:

- Complete Response: clinical and radiologic fistula closure, with no external output
- Partial Response: ≥50% reduction in output compared to baseline
- Failure: ≤50% reduction in output or need for surgery

Radiologic closure was independently assessed by two blinded radiologists who were unaware of the intervention timing or clinical data. Inter-reader agreement was verified before final classification of each case. Outcome assessors were blinded to treatment details. Clinical closure was operationalized as the complete absence of fistula drainage for at least 7 consecutive days, absence of fluid collection on provocation (gentle compression test), and unchanged stoma output (if present).

Recurrence of fistula beyond 12 weeks was not systematically tracked in this preliminary study, and this is acknowledged as a limitation. At this pilot phase, patient-reported outcomes such as pain scores or quality of life were not collected, but these will be included in future phases of the project.

## 3. Results

### 3.1. Patient Demographics and Fistula Characteristics

Six patients (four males; two females) with a mean age of 49 years (range 34–68) were enrolled. Etiologies included diverticulitis, blunt trauma, previous abdominal surgery, acute necrotizing pancreatitis and infected mesh removal. All patients had high output enterocutaneous fistula, with output ranging from 200 to 350 mL/day.

**Figure 1:**
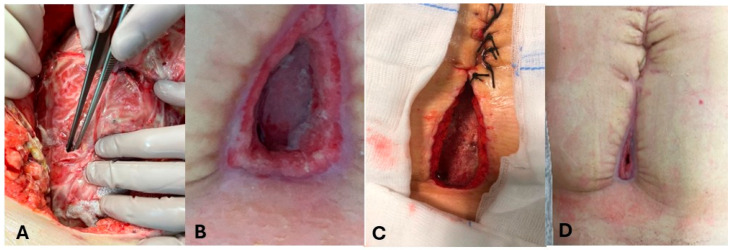
Evolution of enterocutanous fistula in patient 4 (P4). (**A**): Intraoperative findings in P4. (**B**–**D**): healing process of enterocutaneous fistula in P4.

- One patient (P1) demonstrated an enterocutaneous fistula, extending between the left colon and the skin of the left flank, due to anastomotic leak after sigmoidectomy for acute diverticulitis.
- One patient (P2) underwent emergency splenectomy and ileocolic resection for major blunt trauma and developed a fistula extending from the site of anastomotic leakage to the drain insertion.
- Two patients (P3-P4) demonstrated enterocutaneous fistulas following colorectal surgery and gynecological surgery. Intraoperative findings, evolution, and healing process of enterocutaneous fistula in P4 are shown in Figure 1.

**Figure 2:**
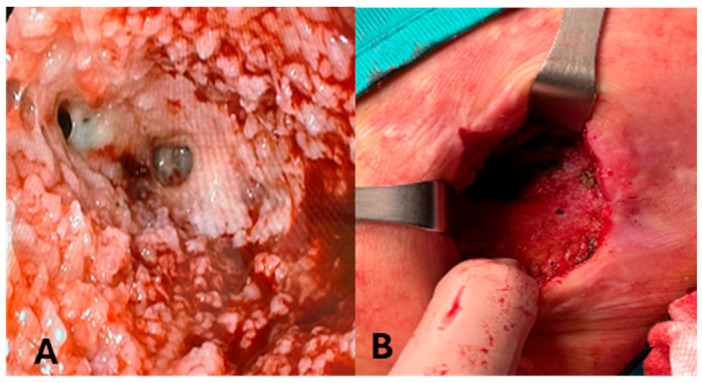
Evolution of enterocutaneous fistula in patient 5 (P5). (**A**): Intraluminal image of colocutaneous fistula in P5. (**B**): P5 after complete resolution of enterocutaneous fistula.

One patient (P5) developed an enterocutaneous fistula due to a pressure ulcer and left colon microperforation, caused by a percutaneous abdominal drain (Figure 2). The patient was affected by necrotizing pancreatitis, treated following the step-up approach with multiple surgical interventions for necrosectomy.

**Figure 3:**
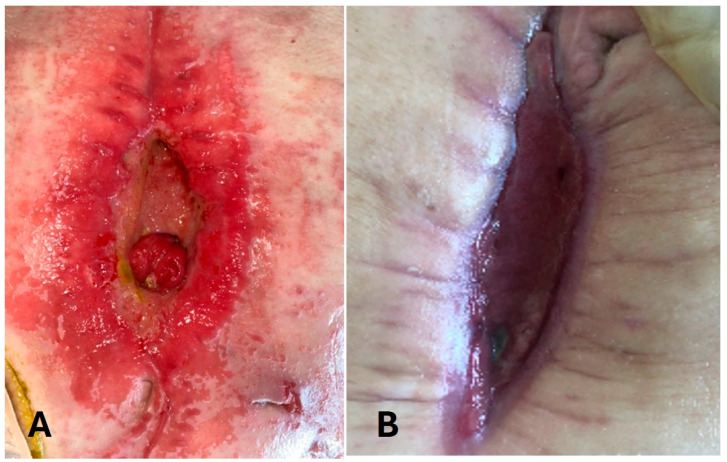
Evolution of enterocutaneous fistula in patient 6 (P6). (**A**): Enterocutaneous fistula at the beginning; (**B**): 3 months after ADSC injection.

One patient (P6) with a chronic mesh infection had a fistula extending from small bowel and periumbilical skin, after removing the infected prosthesis (Figure 3).

### 3.2. Clinical Outcomes

- Three patients (P2-P4-P5) achieved complete closure with no residual output and radiologic resolution at 4 weeks.
- One patient (P1) achieved complete closure with no residual output and radiologic resolution at 12 weeks.
- One patient (P6) had a significant reduction (≥80%) in output, but incomplete closure at 12 weeks.
- One patient (P3) demonstrated no improvement and underwent surgical repair at 6 weeks.

Inflammatory markers normalized in all responders. Albumin level and body weight improved in complete responders by 12 weeks. Quantitatively, mean CRP levels decreased from 5.8 ± 2.1 mg/dL at baseline to 1.1 ± 0.6 mg/dL at 12 weeks (*p* < 0.05), while mean albumin increased from 2.8 ± 0.3 g/dL to 3.6 ± 0.4 g/dL. Body weight improved by an average of 2.1 ± 0.7 kg among complete responders. Radiologic assessment confirmed complete tract obliteration in four patients and significant lumen narrowing in one partial responder. Inter-reader agreement between the two radiologists was high (κ = 0.89), confirming reproducibility of imaging-based healing assessment. No delayed adverse events, abscesses, or ectopic tissue formation were reported up to 12 weeks. No patient developed fever, abscess formation, allergic reaction or systemic complications related to ADSC administration.

The results are shown in Table 1.

## 4. Discussion

This preliminary case series supports the feasibility and early efficacy of ADSC injection for the treatment of complex postoperative enterocutaneous fistulas, especially in patients unresponsive to standard conservative treatment. Our findings are consistent with preclinical studies demonstrating that ADSCs promote epithelial restitution and reduce inflammation in intestinal injury models through secretion of cytokines such as IL-10, HGF, and VEGF. These biological mechanisms may explain the rapid improvement in output and healing observed in our patients. Moreover, the detailed experimental standardization adopted in this study (closed-system harvest, viability testing, and sterility control) contributes to the reproducibility and translational potential of the results. In our cohort, four out of six patients (66.6%) achieved complete clinical and radiologic closure within 12 weeks, while one experienced significant reduction in output, highlighting the therapeutic promise of ADSC therapy in anatomically complex and surgically hostile cases.

Mesenchymal stem cells (MSCs) are multipotent, adult stem cells capable of self-renewal and differentiation into different cell types, such as adipocytes, osteoblast, myocytes, etc., and they have emerged as powerful tools in regenerative medicine due to their immunomodulatory, anti-inflammatory, and pro-angiogenic properties [13,14].

Numerous clinical studies have also demonstrated how MSCs play a key role in immunoregulation processes, having anti-inflammatory, anti-apoptotic, analgesic, angiogenic and anti-oxidative effects [15,16]. Adipose-derived MSCs, in particular, are appealing given their abundance, ease of harvest, and low donor site morbidity.

MSCs, migrated into injured and inflamed tissues, are able to express their differentiation and coordinate tissue repair processes, increasing neo angiogenesis, recruiting other cells and secreting growth factors and matrix proteins [17].

The use of bone-marrow-derived stem cells (BMDSCs) in various field of medicine has been widely known. In 2001, Zuk et al. first reported that adipose tissue contains a large amount of mesenchymal stem cells [18]. Adipose tissue has clear advantages over bone marrow as a source of mesenchymal stem cells. First, it is abundant in the human body as subcutaneous tissue, and it can be harvested from patients in a less invasive manner. Moreover, the procedure can be easily performed in a day-surgery mode without requiring patient hospitalization [19,20]. ADSCs exert their effects through secretion of growth factors (VEGF, TGF-β, IGF), anti-inflammatory cytokines, and matrix remodeling enzymes. Their paracrine signaling may enhance granulation tissue formation, epithelial regeneration, and local immune modulation.

Another unresolved issue is the best delivery system to allow stem cells to penetrate the tissues and perform their functions. Stem cell products can be prepared as simple suspension or combined with biological products or biomaterials [21].

Stem cell therapy has been demonstrated as safe and has shown promising results in a wide variety of clinical and experimental settings. At present, one of the target diseases of treatment with ADSCs is perianal fistulas in Crohn’s disease [22,23,24]. The exact mechanism of perianal fistula healing by ADSCs remains unknown but may reflect the characteristics of mesenchymal stem cells, including their potential for differentiation [25,26] and their anti-inflammatory and immunomodulatory effects [27], in murine and human models. The ADMIRE-CD trial and subsequent long-term follow-ups have confirmed the safety and efficacy of allogeneic expanded ADSCs (darvadstrocel), leading to regulatory approval in Europe. However, extrapolation of these results to non-IBD, postoperative ECFs remains limited. Based on this knowledge, it was understood how stem cell treatment can also be a safe and effective treatment tool for enterocutaneous fistulas [28,29].

This condition still represents a challenge for surgeons and requires treatments that are received over time and involve numerous medial specialists.

Our study demonstrates that even in challenging scenarios, such as anastomotic leakage and previous mesh infections, a subset of patients can benefit from biologically directed healing. The success in our cohort mirrors previous results from Panes et al. [30], Garcia-Olmo et al. [11] and Dige et al. [31], who noted durable responses with local MSC injection. However, unlike those studies that used expanded allogenic products, we used autologous, non-expanded ADSCs, reducing immunogenicity risk and regulatory barriers. Notably, we employed autologous, non-expanded ADSCs derived from fresh lipoaspirate, circumventing regulatory challenges associated with allogeneic or culture-expanded cell products. This pragmatic approach aligns with the successful model applied by Dige et al. [31] and Potenza et al. [12] in similar contexts. Furthermore, our results echo those of Mizushima et al. [6], who reported resolution in a comparable patient population using autologous SVF.

An important clinical implication of this approach lies in its minimally invasive nature, allowing early biologically directed healing in patients otherwise deemed high risk for reoperation. In our series, patients with fistulas secondary to necrotizing pancreatitis, infected mesh, and prior radiation could be managed without surgery. This opens possibilities for novel triage strategies, particularly in resource-constrained settings or when surgical timing must be deferred.

Nevertheless, challenges persist. The optimal delivery route, volume, and concentration of ADSCs remain undefined. In our study, direct intralesional injection with a 25-gauge needle appeared safe and effective, but may not be generalizable to all fistula types. Other platforms under investigation include ADSCs combined with biological scaffolds or hydrogels, which may enhance cellular retention and efficacy [21]. The observed heterogeneity in response—particularly the lack of complete closure in patients P3 and P6—may reflect multiple factors, including local tissue fibrosis, ongoing infection, reduced ADSC engraftment, or host immune differences. Anatomical factors such as tract length and proximity to contaminated cavities might also have influenced outcomes. The variable ADSC yield among patients, likely due to individual adipose tissue characteristics and donor age, could represent another determinant of treatment efficacy. Standardization of harvest and processing protocols will be essential to minimize this variability.

Radiologically, fistula monitoring remains dependent on CT or MRI, with variable inter-reader reliability. Integration of radiomic biomarkers may in the future provide more objective markers of healing. Radiomics—the high-throughput extraction of quantitative imaging features—could help stratify fistula complexity and predict outcomes post-cell therapy, linking regenerative medicine and surgical decision-making.

Importantly, stem cell therapies still lack unified regulatory and processing standards, which hinders reproducibility. Institutional differences in GMP protocols, patient selection, and cell viability assessments may significantly affect clinical outcomes. Cross-center harmonization and multicentric trials are thus urgently needed.

## 5. Limitations

This study has several limitations. First, the sample size is small, and the lack of randomization and control group precludes definitive conclusions regarding efficacy. Second, despite standardized processing, interpatient variability in ADSC yield and viability may have influenced the healing response. Third, patient-reported outcomes (pain, quality of life) were not collected in this pilot phase but are planned for future studies. Finally, as a single-center experience, generalizability remains limited, and larger multicentric trials are necessary to validate reproducibility and refine patient selection.

## 6. Conclusions

ADSC therapy appears to be a promising, safe, and minimally invasive option for the treatment of postoperative enterocutaneous fistulas that are unresponsive to conventional conservative measures. In this small prospective case series, two-thirds of patients achieved complete fistula closure, with notable improvement in systemic inflammation and nutritional status and no reported adverse events. The findings suggest that ADSCs, through their regenerative and immunomodulatory mechanisms, may provide an alternative or adjunctive therapy in anatomically complex or surgically hostile cases. However, these results must be interpreted within the context of the study’s limitations, including its small sample size, short follow-up period, lack of randomization, and absence of a comparator arm. Further randomized controlled trials are needed to validate these findings, determine optimal dosing and administration protocols, and explore long-term efficacy. Establishing standardized cell processing techniques, delivery matrices, and patient selection criteria will be critical for the broader clinical adoption of ADSC-based regenerative therapies in gastrointestinal surgery.

## Figures and Tables

**Table 1:** Summary of patient characteristics and outcomes.

Patient	Sex	Age	Etiology	24 h Inoutput (mL)	Closure at 1 Month	Closure at 3 Months
**P1**	M	52	Acute Diverticulitis	280	No	Yes
**P2**	M	35	Blunt Abdominal Trauma	250	Yes	Yes
**P3**	M	68	Colorectal Surgery	200	No	Required Surgery
**P4**	F	34	Gynecological Surgery	200	Yes	Yes
**P5**	M	40	Acute Necrotizing Pancreatitis	250	Yes	Yes
**P6**	F	65	Chronic mesh infection	300	No	Improved

## Data Availability

The datasets generated and/or analyzed during the current study are available from the corresponding author on reasonable request.

## Abbreviations

The following abbreviations are used in this manuscript:

MSCs	mesenchymal stem cells
ADSCs	adipose-derived mesenchymal stem cells
BMDSCs	Bone-marrow-derived stem cells
SVF	stromal vascular fraction
ECFs	enterocutaneous fistulas
TPN	total parenteral nutrition
IBD	inflammatory bowel disease
WBC	white blood cells
CT	Computed Tomography
MRI	Magnetic Resonance Imaging
